# Implementing a resilience coach program to support first year housestaff during the COVID-19 pandemic: early pilot results and comparison with non-housestaff sessions

**DOI:** 10.1186/s12913-023-09951-1

**Published:** 2023-08-29

**Authors:** Rebecca E. Stewart, Katherine Wislocki, Courtney B. Wolk, Lisa Bellini, Cecilia Livesey, Kelley Kugler, Nayoung Kwon, Nicholas C. Cardamone, Emily M. Becker-Haimes

**Affiliations:** 1 Department of Psychiatry, Perelman School of Medicine at the University of Pennsylvania, 3535 Market Street, Room 3103, Philadelphia, PA 19104 USA; 2 Leonard Davis Institute of Health Economics, Philadelphia, PA USA; 3 Department of Medicine, Perelman School of Medicine at the University of Pennsylvania, Philadelphia, PA USA; 4 The Acceleration Lab, Center for Health Care Innovation, Perelman School of Medicine at the University of Pennsylvania, Philadelphia, PA USA

**Keywords:** Resident wellness, COVID-19, Psychological first aid, Resiliency

## Abstract

**Background:**

In response to the COVID-19 pandemic, we launched the Penn Medicine Coping First Aid program to provide psychosocial supports to our health system community. Our approach leveraged lay health worker volunteers trained in principles of Psychological First Aid to deliver coaching services through a centralized virtual platform.

**Methods:**

We emailed all (*n* = 408) first year housestaff (i.e., residents and fellows) with an invitation to schedule a session with a resilience coach. We compared the mental health concerns, symptoms, and Psychological First Aid techniques recorded in (*n* = 67) first year housestaff sessions with (*n* = 91) sessions of other employees in the health system.

**Results:**

Between June and November 2020, forty-six first year housestaff attended at least one resilience coaching session. First year housestaff most commonly presented with feelings of anxiety and sadness and shared concerns related to the availability of social support. Resilience coaches most frequently provided practical assistance and ensured safety and comfort to first year housestaff. First year housestaff reported fewer physical or mental health symptoms and held shorter sessions with resilience coaches than non-housestaff.

**Conclusions:**

This work offers insights on how to address psychosocial functioning through low-intensity interventions delivered by lay personnel. More research is needed to understand the efficacy of this program and how best to engage housestaff in wellness and resilience programs throughout training, both during and beyond COVID-19.

The COVID-19 pandemic has exacerbated negative mental health outcomes among first responders, medical students, and frontline healthcare staff across the globe [1–3]. Physician residents and fellows (henceforth housestaff), who are already at risk for chronic stress and lower well-being than the general public, faced compounding challenges while in training during COVID-19 [4]. Housestaff surveyed from March 2020 to December 2021 reported high anxiety, mental distress, and various concerns related to the well-being of their patients, loved-ones, and themselves [5, 6]. The personal impact of COVID-19 may be particularly significant for first year residents and fellows due to the lack of traditional supports, the sudden reassignment to emergency departments, and increased isolation or financial stress [7].

In the last decade, many healthcare organizations have implemented wellness interventions to reduce occupational burnout, a chronic form of work-related stress [8]. A review of these interventions found that work hour limits, self-care training, and meditation practice significantly reduced depersonalization and emotional exhaustion among resident physicians [9]. Promisingly, as of 2017, most hospitals in the United States reported offering some form of a stress-management program to their employees [10]. The onset of COVID-19 introduced a barrage of acute stressors that necessitated the provision of more responsive supports to frontline staff. Since March 2020, many healthcare institutions have strengthened their staff wellness infrastructure and introduced resilience-building programs previously employed in disaster situations [5, 11]. The Penn Medicine Coping First Aid (CFA) program is one such support service that was developed between March and June 2020 and made available to all 44,254 Penn Medicine employees, including 1,475 housestaff. Existing wellness programs were already implemented as part of the residency curricula; however the CFA program was specifically designed to provide coping resources to protect against the distress associated with the COVID-19 pandemic.

The CFA program was informed by Psychological First Aid (PFA) techniques, which include the provision of non-intrusive support, a thorough needs evaluation, and linkages to further care. Although psychosocial interventions modeled on PFA have been employed in a range of disaster contexts and populations (Bisson and Lewis 2009), including frontline healthcare workers (Hooper et al. 2021), evidence for its general effectiveness is not yet established [12]. Despite this, PFA was a suitable framework for the COVID-19 response for its ability to be administered remotely [13], be rapidly deployed by trained lay-persons [14], and be adaptable to the shifting context of the crisis [15].

Preliminary results show that those who engaged with the CFA program were highly satisfied and found the services they received beneficial [16]. In the current study, we describe session data from the cohort of housestaff who entered residency or fellowship in June 2020. We report the rate of program uptake, the most commonly recorded concerns, symptoms, and PFA techniques used in sessions, and where individuals were referred. In an effort to iteratively improve and tailor the CFA program to better support the specific needs and concerns of first year housestaff, we compare session characteristics of first year housestaff and other Penn Medicine personnel.

## Methods

In the present intervention, volunteer Penn Medicine employees were trained in therapeutic micro-skills based on PFA [17] and readied to provide a number of therapeutic strategies in virtual, one-on-one virtual sessions, including, active listening and emotional support, promoting client self-efficacy, and improving problem-solving abilities. Volunteers were trained with self-paced online webinars, role-playing sessions with feedback, and session guides. These lay mental health providers (henceforth resilience coaches) followed standard protocols for managing emergency situations and linking individuals to appropriate supports. After each session, resilience coaches systematically tracked participant concerns (e.g., sleep difficulties, loneliness, fear of contracting COVID-19), symptoms (e.g., behavioral, emotional, physical, cognitive), and techniques employed during that session (e.g. relaxation techniques, developing an action plan, ensuring physical safety). Items on these tracking sheets were adapted from established PFA checklists available through the World Health Organization [18, 19]. Session data was directly recorded into REDCap, a secure, web-based electronic data capture and management platform developed by the REDCap Consortium [20]. Licensed mental health professionals reviewed session data, provided feedback, and ensured protocol fidelity during weekly meetings with resilience coaches.

### Participants

In June 2020, 408 first year housestaff were introduced to the CFA program during orientation and sent an electronic letter with information about the program and a link to confirm an appointment. In an effort to increase engagement, housestaff were automatically scheduled appointments with resilience coaches and had to opt-out. CFA sessions were scheduled and administered via a secure telemedicine platform which automatically recorded audio, session length, and session type (i.e. new or follow-up). The length of telehealth visits has been used as a proxy for service engagement and included in exploratory analyses in other contexts [21, 22]. We analyzed sessions held between June and November 2020. All other Penn Medicine employees encountered the CFA program through general internal messaging. We used a convenience sample of 91 sessions attended by these personnel during the same timeframe to compare housestaff and non-housestaff sessions. Data were deidentified and procedures were approved by the Institutional Review Board of the University of Pennsylvania.

### Data analysis

We describe session length, number of follow-up sessions, endorsed concerns and symptoms, techniques utilized by coaches in sessions, and referral destinations for housestaff and non-housestaff sessions. We use two-way ANOVA and a series of chi-square analyses to compare session characteristics across client types. Given the number of these exploratory analyses, we apply a conservative α level of 0.001.

## Results

### Participants and sessions

Of the 408 first year housestaff contacted in June 2020, 46 (11.27%) made an appointment with a resilience coach and attended 67 sessions for an average of 34.44 min. The duration of first year housestaff sessions were significantly shorter than non-housestaff sessions (*F* = 26.93, 34.25 vs. 47.33 min, *p* < 0.001). In addition, the group of non-housestaff were composed of more follow-up patients relative to the first year housestaff group (*X*^2^ = 22.30, 27% vs. 67%, *p* < 0.001).

### Participant concerns

Most first year housestaff reported concerns about availability of social support (58.97%). A portion of first year housestaff expressed concerns about finances (6.41%), history of prior trauma and loss (6.41%) and past or preexisting trauma (incl. psychological problems and substance abuse problems; 6.41%). There were no significant differences in presenting concerns between the first year housestaff and other personnel (see Table 1 for the full list of presenting concerns).

**Table 1:** Presenting concerns, symptoms, and techniques employed during CFA sessions

	First Year Housestaff (*N* = 67)	Other personnel (*N* = 91)	χ	*p*
**Concern**				
Availability of social support	58.97%	48.60%	1.555	.2123
History of prior trauma and loss	6.41%	10.28%	0.436	.5093
Past or preexisting trauma/psychological problems/substance abuse problems	6.41%	5.61%	0.000	1
Financial concerns	6.41%	0.00%	4.823	.0281
Concerns about ongoing threat	3.85%	17.76%	7.057	.0079
Concerns about safety of loved one(s)	3.85%	12.15%	2.956	.0856
Extreme guilt or shame	3.85%	11.21%	2.373	.1234
Nature and severity of disaster experiences	3.85%	8.41%	0.889	.3458
Other specific concerns	3.85%	7.48%	0.513	.4738
Loved one(s) diagnosed or hospitalized with COVID-19 or dead	3.85%	0.00%	2.120	.1454
Physical/mental health illness and medication(s)	2.56%	16.82%	8.091	.0044
Living arrangements	2.56%	2.80%	0.000	1
Other concerns	1.28%	3.74%	0.312	.5766
Has been diagnosed with COVID-19	1.28%	0.00%	0.025	.8736
Prior alcohol or drug use	1.28%	0.00%	0.025	.8736
Death of a family member or friend	0.00%	5.61%	2.910	.0880
Thoughts of harming self or others	0.00%	3.74%	1.475	.2245
Concerns about child/adolescent	0.00%	1.87%	0.244	.6212
Spiritual concerns	0.00%	1.87%	0.244	.6212
Displaced from home	0.00%	0.93%	0.000	1
Lost job or school	0.00%	0.93%	0.000	1
Assisted with rescue/recovery	0.00%	0.00%	–	–
At risk of losing own life	0.00%	0.00%	–	–
Concerns over developmental impact	0.00%	0.00%	–	–
Disaster-related losses	0.00%	0.00%	–	–
Has physical/emotional disability	0.00%	0.00%	–	–
Medication stabilization	0.00%	0.00%	–	–
**Symptom**				
**Behavioral**				
Isolation/withdrawal	2.56%	3.74%	0.001	.9801
Maladaptive coping	1.28%	6.54%	1.879	.1704
Other behavioral	1.28%	2.80%	0.036	.8486
Excessive drug, alcohol, or prescription drug use	1.28%	0.00%	0.025	.8736
Separation anxiety	0.00%	0.93%	0.000	1
Extreme disorientation	0.00%	0.00%	–	–
High risk behavior	0.00%	0.00%	–	–
Regressive behavior	0.00%	0.00%	–	–
Violent behavior	0.00%	0.00%	–	–
**Cognitive**				
Difficulty concentrating	2.56%	10.28%	3.015	.0825
Intrusive thoughts or images	2.56%	8.41%	1.812	.1783
Other cognitive	0.00%	2.80%	0.813	.3673
Difficulty making decisions	0.00%	1.87%	0.244	.6212
Distressing dreams or nightmares	0.00%	0.93%	0.000	1
Difficulty remembering	0.00%	0.00%	–	–
Inability to accept/cope with death of loved one(s)	0.00%	0.00%	–	–
Preoccupation with death/ destruction	0.00%	0.00%	–	–
**Emotional**				
Feeling anxious, fearful	16.67%	41.12%	11.535	.0007*
Sadness, tearful	6.41%	20.56%	6.156	.0131
Feelings of guilt or shame	5.13%	16.82%	4.825	.0281
Acute stress reactions	5.13%	12.15%	1.890	.1692
Other emotion	3.85%	8.41%	0.889	.3458
Despair, hopeless	0.00%	13.08%	9.250	.0024
Irritability, anger	0.00%	5.61%	2.910	.0880
Feeling emotionally numb, disconnected	0.00%	2.80%	0.813	.3673
Acute grief reactions	0.00%	1.87%	0.244	.6212
**Physical**				
Sleep difficulties	5.13%	24.30%	10.833	.0010*
Fatigue/exhaustion	3.85%	9.35%	1.332	.2485
Difficulty eating	1.28%	6.54%	1.879	.1704
Other physical symptom	0.00%	4.67%	2.180	.1398
Headaches	0.00%	3.74%	1.475	.2245
Stomachaches	0.00%	3.74%	1.475	.2245
Worsening of health conditions	0.00%	1.87%	0.244	.6212
Chronic agitation	0.00%	0.00%	–	–
**Technique**				
**Safety and Comfort**				
Asked about immediate needs	48.72%	50.47%	0.007	.9314
Encouraged social engagement	41.03%	31.78%	1.303	.2536
Took steps to ensure immediate physical safety	26.92%	38.32%	2.142	.1433
Attended to physical comfort	14.10%	11.21%	0.131	.7172
Assisted with concern over separation from loved one	12.82%	7.48%	0.921	.3371
Attended to traumatic grief	2.56%	1.87%	0.000	1
Gave information about the disaster/risks	2.56%	1.87%	0.000	1
Assisted with acute grief reactions	1.28%	3.74%	0.312	.5766
Attended to spiritual issues regarding death	0.00%	0.93%	0.000	1
Assisted after death of loved one	0.00%	0.00%	–	–
Helped with confirmation of death to child	0.00%	0.00%	–	–
Helped with talking to children about death	0.00%	0.00%	–	–
Provided information about funeral issues	0.00%	0.00%	–	–
**Practical Assistance**				
Helped to develop an action plan	61.54%	76.64%	4.226	.0398
Helped to identify most immediate need(s)	60.26%	66.36%	0.486	.4856
Helped to clarify need(s)	56.41%	65.42%	1.191	.2751
Helped with action to address the need	38.46%	57.94%	6.092	.0136
**Connection with Social Supports**				
Discussed support seeking and giving	30.77%	50.47%	6.393	.0115
Helped problem-solve obtaining/giving social support	23.08%	42.06%	6.416	.0113
Modeled supportive behavior	14.10%	26.17%	3.256	.0712
Facilitated access to primary support persons	3.85%	20.56%	9.401	.0022
Engaged youth in activities	0.00%	0.00%	–	–
**Stabilization**				
Used grounding or relaxation technique	5.13%	9.35%	0.624	.4297
Gathered information for referral for stabilization	2.56%	4.67%	0.124	.7247
Helped with stabilization	0.00%	11.21%	7.597	.0058

^*^Significant at .001 level

### Participant symptoms

First year housestaff reported feelings of anxiety (16.67%) and sadness (6.41%) during their resilience coach sessions. A number of first year housestaff reported sleep difficulties (5.13%) and fatigue or exhaustion (3.85%). Relative to non-housestaff, first year housestaff had fewer reports of anxiety and fearfulness (*X*^2^ = 14.22, 17% vs. 41%, *p* < 0.001) and sleep difficulties (*X*^2^ = 10.83, 5% vs. 24%, *p* = 0.001) during sessions (see Table 1 for the full list of reported symptoms).

### Session protocol

Resilience coaches most commonly offered practical assistance to first year housestaff, such as: helping to develop an action plan (61.54%), helping to identify their immediate needs (60.26%) and helping to clarify their needs (56.41%). First-year housestaff were also commonly asked about their immediate needs (48.72%), encouraged to have social engagement (41.03%) and had steps taken to ensure their physical safety (26.92%). There were no significant differences in techniques used by resilience coaches in sessions with first year housestaff and non-housestaff (See Table 1 for the full list of session techniques).

### Referrals

Approximately half (53.73%) of first year housestaff were connected with other services. Among those referred, resilience coaches most commonly (77.77%) connected them to internal resources (e.g. other services on Penn COBALT, Penn Medicine Together) and external professional mental health services (33.33%). We did not find any significant differences in the rate of referral or where individuals were referred between first year housestaff and non-housestaff.

## Discussion

The Penn Medicine CFA program was developed and implemented in a large urban health system within 90 days of the COVID-19 outbreak in the U.S., providing free, timely psychosocial support to all Penn Medicine employees. We find that most of the cohort of first year housestaff that we solicited did not engage with the program: only one in ten made an appointment with a resilience coach. We find that housestaff who attended a resilience coach session presented with only slight differences in emotional symptoms from the comparison group of non-housestaff. This finding suggests that universal wellness initiatives in healthcare systems may be appropriate, and that perhaps only minimal tailoring is needed to target housestaff. Resilience coaches recorded fewer physical or mental health symptoms and held shorter sessions with first year housestaff compared to non-housestaff. It is unclear whether first year housestaff experienced fewer symptoms prior to beginning training despite COVID-19 or if they felt uncomfortable reporting symptomology in an employer-provided program, despite assurances of confidentiality. To account for these possibilities, resilience coaches were given explicit guides to facilitate sessions with individuals that did not present with specific concerns in the following year’s cohort. This refinement shifted the emphasis to proactive tactics, such as a guided exploration of the wellness services in the organization and sharing psychoeducational resources.

The Penn CFA program is among a number of supportive initiatives that sought to improve the wellbeing of healthcare workers at the onset of the COVID-19 pandemic [23, 24]. Many studies detailing the efficacy of these interventions (or lack thereof) are still forthcoming. Nonetheless, multiple reports continue to document the staggering rates of psychological distress among healthcare workers during COVID-19. A recent meta-review reports that the prevalence of psychophysiological stress among healthcare workers globally to be 37.7%, a rate significantly higher than the general public [25]. In North America, rates of anxiety and depressive symptoms among healthcare workers are as high as 14.8% and 18.7%, respectively, nearly four times the rate among the public before the pandemic [26]. We echo the call for more rigorous research of wellness initiatives for healthcare workers to counteract these effects [27].

This study is limited by the lack of systematic information on the composition of the non-housestaff group. Session notes reveal that this group may be composed of administrators, human resources staff, clinical nurse managers, intake specialists, second-year residents, and medical students, however due to confidentiality protocols, most individuals did not disclose their position within the organization. Unlike the first year housestaff, individuals in the non-housestaff group were not directly solicited and instead encountered the CFA program passively through internal messaging, potentially leading to sample bias. The results suggest that the non-housestaff group was composed of individuals in more severe distress than the group of first year housestaff given their higher incidence of symptoms, longer session length, and higher follow-up rate. In addition, we did not collect information from housestaff who opted out of their resilience coach session, so reasons for declining to participate are unknown. The rate of reported symptoms and concerns is that among housestaff who participated in the program, not from the cohort as a whole. Future research on the CFA program should measure psychological wellbeing before and after the intervention period and employ follow-up surveys to track subsequent service utilization in the entire cohort. Of note, the CFA program was and continues to be staffed by volunteers. Consequently, it is unclear how such a program would scale in other settings as implementation support of new interventions can vary across organizational cultures and communities [28, 29]. Subsequent programming should explore non-volunteer models, such as peer support models, which have shown promise in healthcare settings [11, 30].

Although we are unable to gauge the program’s effectiveness, the data described in this study informed ongoing resilience coach training and better equipped coaches to support subsequent cohorts of residents. In line with guidelines described elsewhere [16], we recommend that other organizations who seek to develop initiatives like CFA take a similar approach of iterative testing and refinement to best support healthcare workers during COVID-19 and beyond.

## Conclusions

This study is one of the first to document the physical and psychological well-being of a cohort of first year housestaff and the implementation of a low-intensity interventions delivered by lay personnel in the early months of the COVID-19 pandemic in the United States. Although future research will need to evaluate the outcomes of this program, Penn Coping First Aid shows promise as a flexible model for delivering psychosocial support to healthcare workers during times of crisis.

## Data Availability

The datasets used and/or analyzed during the current study are available from the corresponding author on reasonable request.

## Abbreviations

CFA: Coping First Aid
PFA: Psychological First Aid
